# Enrichment of *Marinobacter* sp. and Halophilic Homoacetogens at the Biocathode of Microbial Electrosynthesis System Inoculated With Red Sea Brine Pool

**DOI:** 10.3389/fmicb.2019.02563

**Published:** 2019-11-07

**Authors:** Manal F. Alqahtani, Suman Bajracharya, Krishna P. Katuri, Muhammad Ali, Ala’a Ragab, Grégoire Michoud, Daniele Daffonchio, Pascal E. Saikaly

**Affiliations:** ^1^King Abdullah University of Science and Technology, Water Desalination and Reuse Center, Biological and Environmental Science and Engineering Division, Thuwal, Saudi Arabia; ^2^King Abdullah University of Science and Technology, Red Sea Research Center, Biological and Environmental Science and Engineering Division, Thuwal, Saudi Arabia

**Keywords:** Red Sea brine pool, microbial electrosynthesis, metagenome-assembled genome, marinobacter, halophilic homoacetogens

## Abstract

Homoacetogens are efficient CO_2_ fixing bacteria using H_2_ as electron donor to produce acetate. These organisms can be enriched at the biocathode of microbial electrosynthesis (MES) for electricity-driven CO_2_ reduction to acetate. Studies exploring homoacetogens in MES are mainly conducted using pure or mix-culture anaerobic inocula from samples with standard environmental conditions. Extreme marine environments host unique microbial communities including homoacetogens that may have unique capabilities due to their adaptation to harsh environmental conditions. Anaerobic deep-sea brine pools are hypersaline and metalliferous environments and homoacetogens can be expected to live in these environments due to their remarkable metabolic flexibility and energy-efficient biosynthesis. However, brine pools have never been explored as inocula for the enrichment of homacetogens in MES. Here we used the saline water from a Red Sea brine pool as inoculum for the enrichment of halophilic homoacetogens at the biocathode (−1 V vs. Ag/AgCl) of MES. Volatile fatty acids, especially acetate, along with hydrogen gas were produced in MES systems operated at 25 and 10% salinity. Acetate concentration increased when MES was operated at a lower salinity ∼3.5%, representing typical seawater salinity. Amplicon sequencing and genome-centric metagenomics of matured cathodic biofilm showed dominance of the genus *Marinobacter* and phylum Firmicutes at all tested salinities. Seventeen high-quality draft metagenome-assembled genomes (MAGs) were extracted from the biocathode samples. The recovered MAGs accounted for 87 ± 4% of the quality filtered sequence reads. Genome analysis of the MAGs suggested CO_2_ fixation via Wood–Ljundahl pathway by members of the phylum Firmicutes and the fixed CO_2_ was possibly utilized by *Marinobacter* sp. for growth by consuming O_2_ escaping from the anode to the cathode for respiration. The enrichment of *Marinobacter* sp. with homoacetogens was only possible because of the specific cathodic environment in MES. These findings suggest that in organic carbon-limited saline environments, *Marinobacter* spp. can live in consortia with CO_2_ fixing bacteria such as homoacetogens, which can provide them with fixed carbon as a source of carbon and energy.

## Introduction

Homoacetogens are metabolically diverse anaerobic bacteria capable of growing autotrophically on H_2_-CO_2_ and heterotrophically on a wide range of sugars, alcohols, aromatics and one-carbon compounds, producing acetate as the end-product (Diekert and Wohlfarth, 1994). Homoacetogens are important contributors in carbon cycling in the subsurface and marine deep biosphere (Pedersen et al., 2008; Griebler and Lueders, 2009; Heuer et al., 2009). They are among the most phylogenetically diverse bacterial functional groups (Tanner and Woese, 1994) with almost 100 identified species that are phylogenetically classified in 23 different genera (Drake et al., 2008; Schuchmann and Müller, 2014). However, their diversity remains underexplored due to their widespread distribution and multiple routes of metabolism (Drake et al., 2008; Lever, 2012). In particular, their diversity in extreme environments, such as hypersaline habitats, is poorly studied.

Extreme marine environments host unique microbial communities that survive the harsh physiochemical conditions through specialized mechanisms (Dopson et al., 2016; Poli et al., 2017). The metabolic capabilities of extremophiles have a large application potential and hence, it is highly attractive to investigate these organisms to exploit for new metabolites and metabolisms (Raddadi et al., 2015). Deep sea brine pools are hypersaline (up to 25%) environments with low oxygen < 0.2 mg/L (Gurvich, 2006; Shehab et al., 2017) and absence of light. In the Red Sea there are present several brine pools containing mineralized brines with metalliferous deposits, which have been generated from the dissolution of Miocene rocks eventually combined with hydrothermal activities in deep sea floor (Behzad et al., 2016). In spite of the inhospitable conditions, Red Sea brine pools support a highly specialized microbial diversity, especially along the steep halocline that occurs along the seawater-brine interface (Wang et al., 2013). While culturing such microorganisms in the laboratory is difficult, genomic and metagenomic approaches have revealed several metabolic pathways occurring in brine pools (Bougouffa et al., 2013; Behzad et al., 2016). Metagenomes have revealed autotrophic metabolisms occurring in the microbial assemblages of the Red Sea brine pools (Wang et al., 2013; Behzad et al., 2016). Further, CO_2_ reducers are abundant in deep-sea brine waters (Wang et al., 2013; Poli et al., 2017; Merlino et al., 2018). Even though highly specialized homoacetogens can be expected to live in the deep sea brine pools, due to their remarkable metabolic flexibility and energy-efficient biosynthesis (Lever, 2012; Oren, 2012), no studies specifically explored their occurrence in the Red Sea.

Microbial electrosynthesis (MES) is a sustainable biotechnology for the conversion of the greenhouse gas CO_2_ to useful chemicals, mainly methane and acetate. In MES, water oxidation at the abiotic anode produces protons and electrons which are transported to the cathode where microbial-catalyzed CO_2_ reductions to methane [hydrogenotrophic methanogenesis (Alqahtani et al., 2018)] and/or acetate [homoacetogenesis (Bian et al., 2018)] occur. The cathode serves as the reducing equivalent (directly or indirectly via H_2_ evolved at the electrically polarized cathode) for the reduction of CO_2_ to acetate and/or methane.

Several homoacetogenic species can be enriched in MES from anaerobic mixed cultures that are generally obtained from conventional anaerobic sludge sources (Marshall et al., 2017). While inocula from extreme environments, including Red Sea brines, have been tested to enrich electrochemically-active bacteria on the anode of microbial electrochemical systems (BESs) (Miceli et al., 2012; Rousseau et al., 2013; Pierra et al., 2015; Shehab et al., 2017), they have never been explored as a source for the enrichment of homoacetogens on MES cathodes. Further, the presence of high levels of metals (Fe, Mn, Zn, Cu, Pb, Co, Ba, Si, Li) as nanoparticles in the brine pool solution may support a unique solid particle-microbial complexes with potential electroactivity (Anselme et al., 2010). This suggests the possibility that certain microbes in brine pools can use the cathode as an electron donor (directly or indirectly via H_2_).

Enriching halophilic homoacetogens directly in MES system, instead of serum vials under H_2_:CO_2_, is advantageous because homoacetogens can be directly selected by the conditions at the cathode. The cathode acts as the electron donor for homoacetogens – by providing electrons either directly or indirectly through H_2_ generated from the hydrogen evolution reaction (HER). This is important as it is not predictable which route (direct, indirect, or both) homoacetogens in the inocula would prefer for taking electrons from the cathode. Furthermore, the use of saline electrolyte in MES system helps to reduce the internal resistance for ions mobility. Another advantage is that at high salinity, homoacetogens may outcompete methanogens growing on H_2_ and CO_2_ because of the high energy demand of methanogens to accumulate organic compatible solutes for osmoregulation, whereas homoacetogens accumulate KCl to establish osmotic balance which is less energy demanding (Oren, 2011; Lever, 2012). Finally, high salinity controls microbial contamination from freshwater sources.

The objective of this study was to use Red Sea brines as inoculum to enrich for halophilic homoacetogens at the cathode of MES operated at a cathode potential of −1 V vs. Ag/AgCl. The biocathode community was characterized using 16S rRNA gene sequencing, metagenomics, and genome reconstruction. In addition, CO_2_ reduction products were characterized at the cathode.

## Materials and Methods

### Inoculum Source and Chemical Characterization

#### Red Sea Brine Pool Sample Collection

Samples of the seawater-brine interface solution and sediment were collected in April 2017 at a depth of ∼360 m from a newly discovered brine pool (Afifi Deep) located at the southern basin of the Red Sea (Duarte et al., under review). Samples were collected using the R/V Thuwal vessel of the Coastal and Marine Resources Core Laboratory at KAUST. The brine water samples were collected using 10 L Niskin water sampling bottles, with water sampling system consisting of 23-bottle Rosette (Shehab et al., 2017; Merlino et al., 2018). The sediments were sampled using a Multi-corer (KC 6 x ø100 mm Model 70.000). Once onboard, the samples were put in a sealed container under a flux of argon to keep the samples anaerobic. They were then stored at 4°C.

#### Chemicals Analyses of the Brine Pool Interface Solution

The concentration of metals in the interface solution was measured by inductively coupled plasma optical emission spectrometry (ICP-OES) (Optima 8300, Perkin Elmer), equipped with a custom designed solid-state charge-coupled device array detector. The total organic carbon (TOC) concentration in the interface solution was measured using an on-line TOC analyzer (TOC-V_CSH_, Shimadzu) as previously described (Shehab et al., 2017). The conductivity and pH were measured using a WTW Multi 3320 meter. The concentration of sulfate, nitrite-nitrogen (${\text{NO}}_{2}^{-}$-N), nitrate-nitrogen (${\text{NO}}_{3}^{-}$-N) were measured using HACH kits (HACH, Loveland, CO, United States) following manufacturer’s instructions.

### Enrichment Phases

**FIGURE 1:**
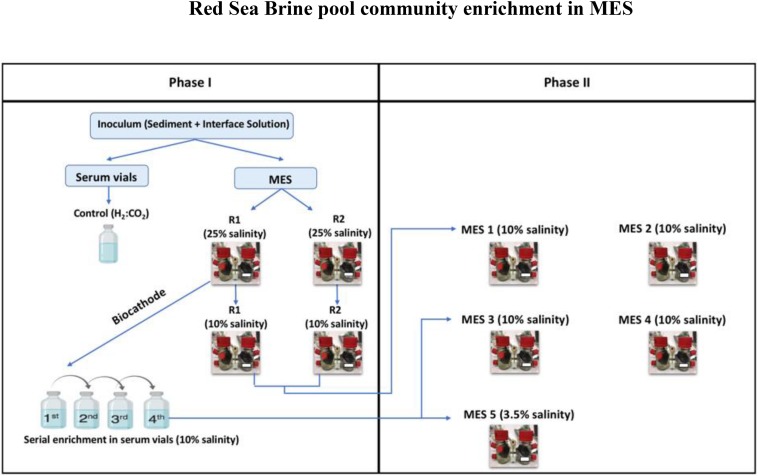
Illustration showing the different enrichment phases.

The enrichment was conducted in two phases as illustrated in Figure 1. In phase I, the brine pool sediment and seawater-brine interface solution were used to inoculate duplicate serum vials, used as control, and duplicate MES reactors designated as R1 and R2. The serum vials were operated with H_2_:CO_2_ (80:20). The vials were incubated at 30°C for 150 days. Every week the vials where flushed with H_2_:CO_2_.

The duplicate MES reactors were operated first for 5-6 months under 25% salinity using the same brine pool interface solution as electrolyte in order to mimic the microbes’ original environment. Then, the brine electrolyte was replaced with 10% saline synthetic media and operated for another 5-6 months.

Before switching the operation of MES reactors to 10% saline synthetic media, biofilm sample from R1 was used to seed serum vials operated under H_2_:CO_2_ (80:20) and 10% saline synthetic media to (i) investigate whether lower salinity can affect the function of biocathode in the MES; and (ii) to enrich biomass to be used as inoculum for MES reactors in phase II (see below). The serum vials underwent four serial enrichments (3-4 weeks each) and once considerable volatile fatty acid (VFA) production was observed the operation of the MES reactors was switched to 10% saline synthetic media.

*Sporomusa ovata* DSMZ growth medium (DSMZ 311) was used as synthetic solvent to dissolve 100 g/L NaCl (10% salinity). The media contains 0.5 g NH_4_Cl, 0.5 g MgCl_2_. 6H_2_O, 0.25 g CaCl_2_. 2H_2_O, 2 mL of FeSO_4_ × 7 H_2_O solution (0.1% w/v in 0.1 N H_2_SO_4_), 1 mL of trace element solution, 1 mL of selenite-tungstate solution, 0.35 g K_2_HPO_4_, 0.23 g KH_2_PO_4_, 2 g of NaHCO_3_, and 10 mL of vitamin solution. The media was prepared as previously described (Möller et al., 1984; Nevin et al., 2010), with slight modifications to meet the salinity requirement and to eliminate any carbon source (like omitting betaine, casitone, yeast extract) and other possible electron acceptors (such as sulfide and cysteine).

In phase II, new sets of MES reactors were operated to develop a secondary biofilm (Liu et al., 2008) at the cathode by adopting two modes of inoculation: (1) inoculating new duplicate MES reactors namely MES 1 and MES 2 by a piece of enriched biocathode (referred to herein as Inoculum_Biofilm) from phase I MES reactors (i.e., R1 and R2) operated at 10% saline synthetic media; and (2) inoculating three new MES reactors (MES 3, MES 4, and MES 5) by enriched culture from phase I serum vial (generation 4; Figure 1) operated under H_2_:CO_2_ (referred to herein as Inoculum_Culture) and 10% saline synthetic media. All new MES reactors were operated under 10% saline synthetic media, except MES 5, which was operated at 3.5% salinity to mimic seawater salinity. The rationale for running MES 5 was to determine the effect of salinity (10 vs. 3.5%) on microbial community composition and product formation.

### MES Reactor Configuration and Operation

Two chambered H-type cells (Adams and Chittenden Scientific Glass) were used as MES reactors. The cathode was made of carbon cloth (3.5 cm width, 6 cm length), and the anode was made of carbon cloth (3.5 cm width, 6 cm length) coated with iridium oxide (Ir_2_O) in one side following the protocol of coating metals on carbon material (Cheng et al., 2006). The current collector for both electrodes was titanium wire. An Ag/AgCl reference electrode, saturated in 3M NaCl (BASi, United States), was placed between the working electrode (cathode) and counter electrode (anode). The anode and cathode chambers were separated by Nafion^®^ 117 membrane (Sigma, United States). The collector gasbag (Calibrate, Inc., United States) was connected to the headspace of the cathode chamber. The total and working volume of each chamber was 150 and 125 mL, respectively. Anolyte chamber was filled by autoclaved seawater-brine interface solution or synthetic saline media, when the reactors were operated under 25% salinity or 10% salinity, respectively. The cathode was poised at −1 V vs. Ag/AgCl using multichannel potentiostat (VMP3 Bio Logic). Since HER occurs at more negative potentials than −0.8 V vs. Ag/AgCl at neutral pH using carbon electrodes due to high overpotentials, we chose −1 V vs. Ag/AgCl to ensure HER at the cathode. Current densities were calculated by normalizing the current to the total surface area (42 cm^2^) of the cathode. All MES reactors were placed on a stirring bar at 120 rpm and operated in a temperature-controlled room (30°C) in the dark.

The reactors were operated in fed-batch mode and CO_2_ was injected through the electrolyte every 5 days and the media was changed every 20-45 days in phase I, whereas the media was changed every 12-15 days of operation in phase II.

### Analytics and Calculations

Gas and electrolyte VFA compositions were analyzed using chromatographic techniques as reported earlier (Ambler and Logan, 2011; Hari et al., 2016). Liquid samples were filtered by 0.2 μm syringe filters and VFAs were measured using a high-performance liquid chromatography (HPLC) equipped with an Aminex column (an HPX-87H Ion Exclusion column; Bio-Rad Laboratories, Inc., Berkeley, CA, United States), with UV detector (210 nm wavelength) and mobile phase 0.005M H_2_SO_4_.

The gas composition (H_2_, N_2_, and CH_4_) in both the cathodic chamber headspace and gasbag was measured using gas chromatography (GC, model 310C, SRI Instruments, United States) with argon as carrier gas. The concentration of CO_2_ was measured using a separate GC (model 310C, SRI Instruments, United States) with a helium carrier gas.

The performance of the MES was evaluated in terms of cathodic columbic efficiency of CO_2_ reduction to product formation (i.e., CH_4_ and/or VFAs in mol/m^2^ cathode/d). The calculation of electron mass balance was estimated using columbic efficiency equations as previously described (Patil et al., 2015; Bajracharya et al., 2016; Alqahtani et al., 2018).

### Scanning Electron Microscopy (SEM)

Biofilm and suspension samples were soaked in 2% glutaraldehyde solution containing phosphate buffer (50 mM, pH 7.0) and stored at 4°C for 2 days. The samples were washed by phosphate buffer, then dehydrated in serial gradual ethanol solutions. The samples were fixed on the aluminum metal stub and coated by Pt/Ir for 30 s at 25 mA current (5 nm thickness) using sputter coating apparatus under argon atmosphere. Scanning electron micrographs were visualized using Quanta 600 FEG and ZIESS (Merlin -61-95).

### Energy-Dispersive X-Ray (EDX) Spectroscopy

The EDX detector on Quanta 600D FEI and ZEISS Merlin 61-95 SEM (accelerating voltage 20 kV and spot-size 6) was switched to determine the elemental composition of the cathode surface. Elemental peaks were identified using the built-in software and the atomic (At)% of each element was analyzed over the scanned area.

### DNA Extraction, Library Preparation, and Sequencing

At the end of phase I and phase II, samples were collected for DNA extraction using the standard protocol for FAST DNA Spin kit for Soil (MP Biomedicals, United States) with the subsequent modifications; 500 μL of sample, 480 μL of sodium phosphate buffer, and 120 μL MT buffer were added to a Lysing Matrix E tube. Bead beating was done at a speed of 6 m/s for 4 x at 40 s each (Albertsen et al., 2015). Gel electrophoresis using Tapestation 2200 and genomic DNA screentapes (Agilent, United States) were performed to examine product size and purity of a subset of DNA extracts. DNA concertation was estimated using Qubit dsDNA HS/BR Assay kit (Thermo Fisher Scientific, United States).

The bacterial and archaeal 16S rRNA gene region V4 sequencing libraries were prepared by a custom protocol based on an Illumina protocol and the libraries were paired-end sequenced (2 bp × 300 bp) on a MiSeq^TM^ (Illumina, United States). Details of library preparation are provided in the Supporting Information.

The shotgun metagenome sequencing library preparation was conducted using a TruSeq^®^ Nano DNA LT Library Prep Kit according to manufacturer instructions (Illumina, United States). Libraries were quantified and normalized to 10 nM. The libraries were pooled and sequenced on HiSeq^TM^ 4000 (Illumina, United States) (2 bp × 150 bp) paired-end platform at KAUST Bioscience Core Laboratory.

### Processing of Sequencing Data

Forward and reverse 16S rRNA amplicon reads were trimmed for quality using Trimmomatic v. 0.32 (Bolger et al., 2014), with the settings SLIDINGWINDOW:5:3 and MINLEN: 225. The trimmed forward and reverse reads were merged using FLASH v. 1.2.7 (Magoč and Salzberg, 2011), with the settings -m 10 -M 250. The trimmed reads were dereplicated and formatted for use in the UPARSE workflow (Edgar, 2013). The dereplicated reads were clustered using the usearch v. 7.0.1090 -cluster_otus command with default settings. Operational taxonomic unit (OTU) abundances were estimated using the usearch v. 7.0.1090 -usearch_global command with -id 0.97 -maxaccepts 0 -maxrejects 0. Taxonomy was assigned using the RDP classifier (Wang et al., 2007), as implemented in the parallel_assign_taxonomy_rdp.py script in QIIME (Caporaso et al., 2010), using -confidence 0.8 and the MiDAS database v. 1.23 (McIlroy et al., 2017), which is a curated database based on the SILVA database, release 123 (Quast et al., 2013). The statistical analysis were performed in R v. 3.5.0 (R Core Team, 2019) through the Rstudio IDE using the ampvis package v.2.3.17 (Albertsen et al., 2015).

The shotgun metagenome sequencing reads obtained in the FASTQ format were processed for quality filtering using the Cutadapt package v. 3.7.1 (Martin, 2011). The trimmed reads were assembled using SPAdes v. 3.11.1 (Bankevich et al., 2012). The sequencing reads were mapped back to the assembly using Burrows-Wheeler Aligner (BWA, v. 0.7.15-r1142-dirty; Li and Durbin, 2010) to generate coverage files for metagenomics binning. The coverage files were converted to the sequence alignment/map (SAM) format using samtools version 1.3.1 (Li et al., 2009).

The metagenome-assembled genomes (MAGs) were recovered from assembled scaffolds based on sequence composition, differential coverage and read-pair linkage by employing the CONCOCT (Alneberg et al., 2014) program available within anvi’o software version 5.2 (Eren et al., 2015). The MAGs were refined manually by following the instructions provided on the anvi’o website^1^ (retrieved on December 2, 2018). Completeness and contamination of MAGs were assessed using CheckM v. 1.0.9 (Parks et al., 2015). Subsequenty, the MAGs were annotated using Prokka version 1.13 (Seemann, 2014). The annotated genome assemblies (GFF3 format produced by Prokka) were processed by Roary version 3.11.2 (Page et al., 2015) for comparative genome analysis. The MAGs were further annotated with GhostKOALA (KEGG Orthology And Links Annotation) annotation tool for K number assignment of KEGG GENES (Kanehisa et al., 2016) and were used for KEGG Mapper to search against KEGG pathway maps. Furthermore, the MAGs were classified taxonomically using the protein phylogeny in the Genome Taxonomy Database (Parks et al., 2018) with the gtdbtk software (version 0.1.3) using *“classify_wf”* command. The MAGs and closely related genomes downloaded from the National Center for Biotechnology Information (NCBI) were imported in anvi’o to create a set of concatenated ribosomal proteins using a set of 49 ribosomal proteins collection from Campbell et al. (2011). The anvi’o command “*anvi-get-sequences-for-hmm-hits*” was used with *-concatenate* flag to generate concatenated amino acid sequences. The phylogenetic tree was computed by MEGA7 using the concatenated amino acid sequences by applying neighbor-joining statistical method (Kumar et al., 2016). Branch node support values were calculated by performing 1000 bootstrap iterations and using the Poisson model.

## Results

### Long Term Enrichment in MES (Phase I) Under Cathodic Condition

#### Current Densities in R1 and R2

**FIGURE 2:**
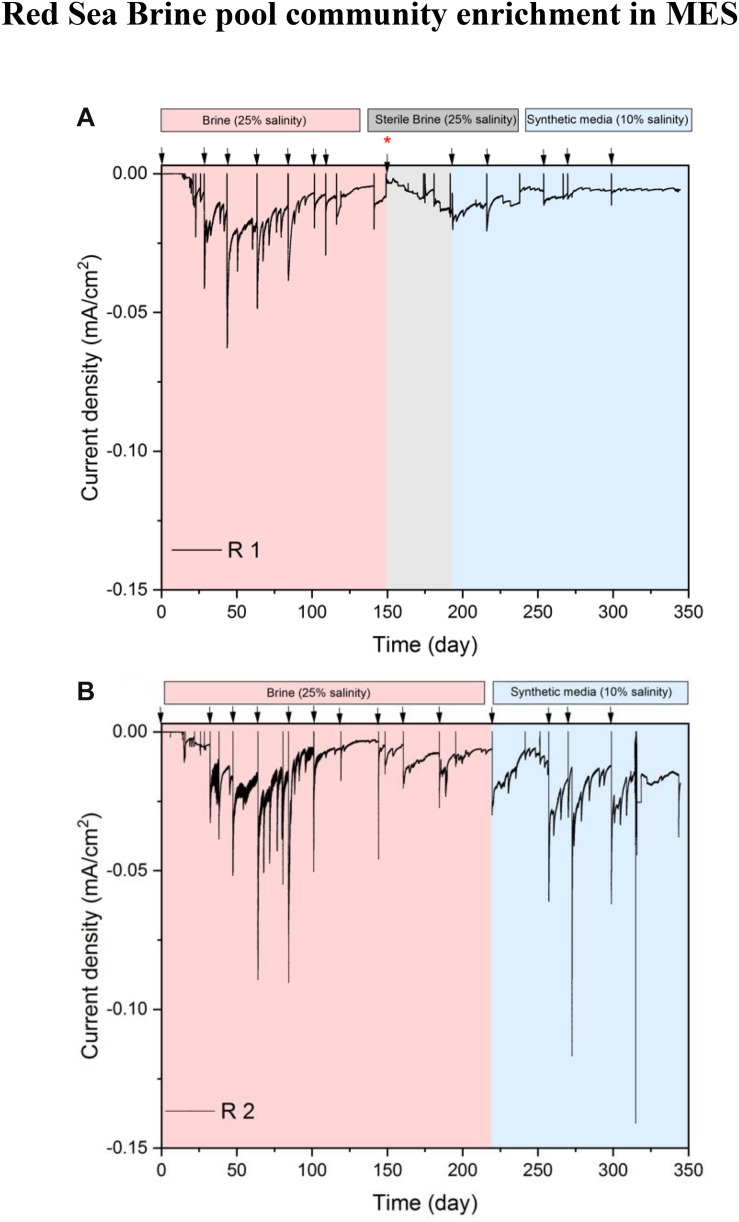
Chronoamperometry profile of phase I. **(A)** Current density of MES reactor R1, which was subjected to three conditions: brine pool solution (25% salinity), sterilized brine pool solution (25% salinity) and synthetic saline media (10% salinity). **(B)** Current density of MES reactor R2, which was subjected to two conditions: brine pool solution (25% salinity) and synthetic saline media (10% salinity). Arrows indicate the time of batch change. The red asterisk on day 150 indicates when biocathode from R1 was transferred to a new MES reactor containing sterile brine pool solution (25% salinity) and a piece of biocathode from R1 was introduced into a serum vial (see Figure 1) containing synthetic saline media (10% salinity). Also, a piece of biocathode from R1 was taken on day 150 for SEM.

Cathodic condition in the MES was used as a selective pressure for the enrichment of halophilic chemolithoautotrophs from brine solution. MES reactors R1 and R2 showed similar behavior in terms of current densities at 25% salinity (Figure 2). The reductive current density started to increase reaching the highest value (−0.025 mA/cm^2^) between days 50 and 70. Until day 70, the MES contained brine pool solution along with brine pool sediment at the cathode chamber to provide more diversity in the microbial inoculum. During that period, the reduction of metal ions, which were abundant in the brine pool (Supplementary Table S2), most probably also contributed to the cathodic current in the MES. After day 70, the sediment was completely removed from the cathode chamber, and after 100 days of operation, the current densities in both MES reactors were in decreasing trend (minimum −0.010 mA/cm^2^) indicating a decline in the reductive activity of the cathode. Lower metal ions present in the catholyte after removing the sediment might have contributed to this drop in current consumption.

On day 150, the biocathode from R1 was placed in a new MES reactor containing filter (0.2 μm)-sterilized brine pool solution to investigate whether the biofilm on the cathode or suspended cells were responsible for current consumption. The current density had an increasing trend with sterile brine pool catholyte, but the highest current density remained at a similar level as the current densities observed in R2 between 150 and 180 days. The current densities in R1 during the sterile brine batch were at a similar level to the previous batches after removing sediment (i.e., days 70–150). These results suggest that suspended microorganisms were not responsible for current consumption. In addition, there was no visible turbidity in the electrolyte of R1 and R2, further supporting that the biological activity of MES was mainly at the biocathode. At the end of the experiment we filtered the whole catholyte solution (125 mL) onto 0.22 μm filters for DNA extraction. The DNA concentration was below the detection limit and no amplification was detected with polymerase chain reaction.

**FIGURE 3:**
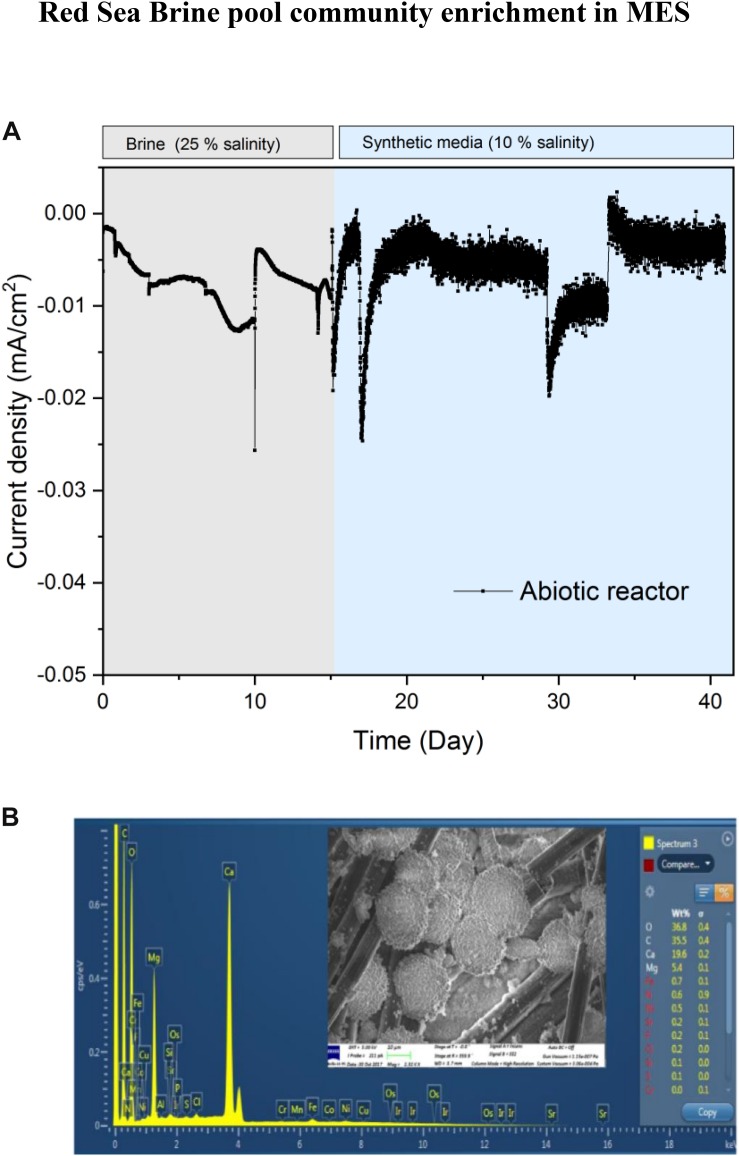
**(A)** Current density profile of abiotic MES reactor operated at −1.0 V vs. Ag/AgCl cathode potential with sterile brine electrolyte (25% salinity) and synthetic saline media (10% salinity) as electrolyte. **(B)** EDX analysis of abiotic carbon felt cathode of the control experiment with sterile brine pool solution (25% salinity). The inset represents SEM image showing salt deposits on the cathode.

To test the response of the microbiome at different salinity conditions, we switched the MES electrolytes in R1 and R2 to 10% salinity using synthetic saline media. However, to determine if lower salinity might affect the performance of biocathodes in R1 and R2, a small piece of biocathode was taken from R1 on day 150 to inoculate a serum vial containing synthetic saline media (10% salinity) and then subjected to incubation under H_2_:CO_2_ (80:20). The results of serum vials showed considerable VFA production suggesting that the cathodic biofilm can adapt to lower salinity and the electrolytes in R1 and R2 were switched to 10% salinity using synthetic saline media. After switching to 10% salinity, the current density in R1 and R2 slightly decreased due to the lowering of electrolyte conductivity from 223 mS/cm (brine pool solution) to 105 mS/cm (synthetic media). In addition, the brine pool solution was acidic (pH 5.85), whereas the pH of the synthetic saline media was neutral. The slight acidity of brine pool solution favors the HER, thus more current consumption was observed when the brine pool solution was used as catholyte. Similar results were observed in the abiotic control reactor, operated under the same conditions as R1 and R2 but with abiotic cathode, where higher current density was observed with sterile brine pool (25% salinity) than with 10% synthetic saline media (Figure 3A).

High amount of ions/metals available in the brine pool solution may affect the current demand of MES reactors due to the electrodeposition of metals at the cathode. This was confirmed by SEM and EDX spectroscopy analysis (Figure 3B) of abiotic cathode operated using sterile brine (25% salinity). SEM analysis of the abiotic cathode was taken after polarization at −1 V vs. Ag/AgCl for 15 days. Precipitation of metal ions and deposits of salt on the carbon felt (Figure 3B inset) were visible, but EDX suggests that the metals were mostly precipitated as carbonate form on the cathode. The carbonate forms of metals usually do not participate in electrochemical CO_2_ reduction, suggesting that CO_2_ reduction in the MES reactors was mainly due to bioelectrochemical activity. This was further supported by the fact that no CH_4_ or VFA production was observed in the abiotic control reactor and hydrogen production (∼2.5 ml H_2_/day) was the main cathodic reaction. Also, the current density in the abiotic reactor (Figure 3A) was significantly lower (Student’s *t*-test; *p* < 0.01) than in MES reactors (Figure 2), further supporting that biofilm community on the cathode was responsible for additional current consumption for CO_2_ reduction.

#### Product Formation in R1 and R2

**FIGURE 4:**
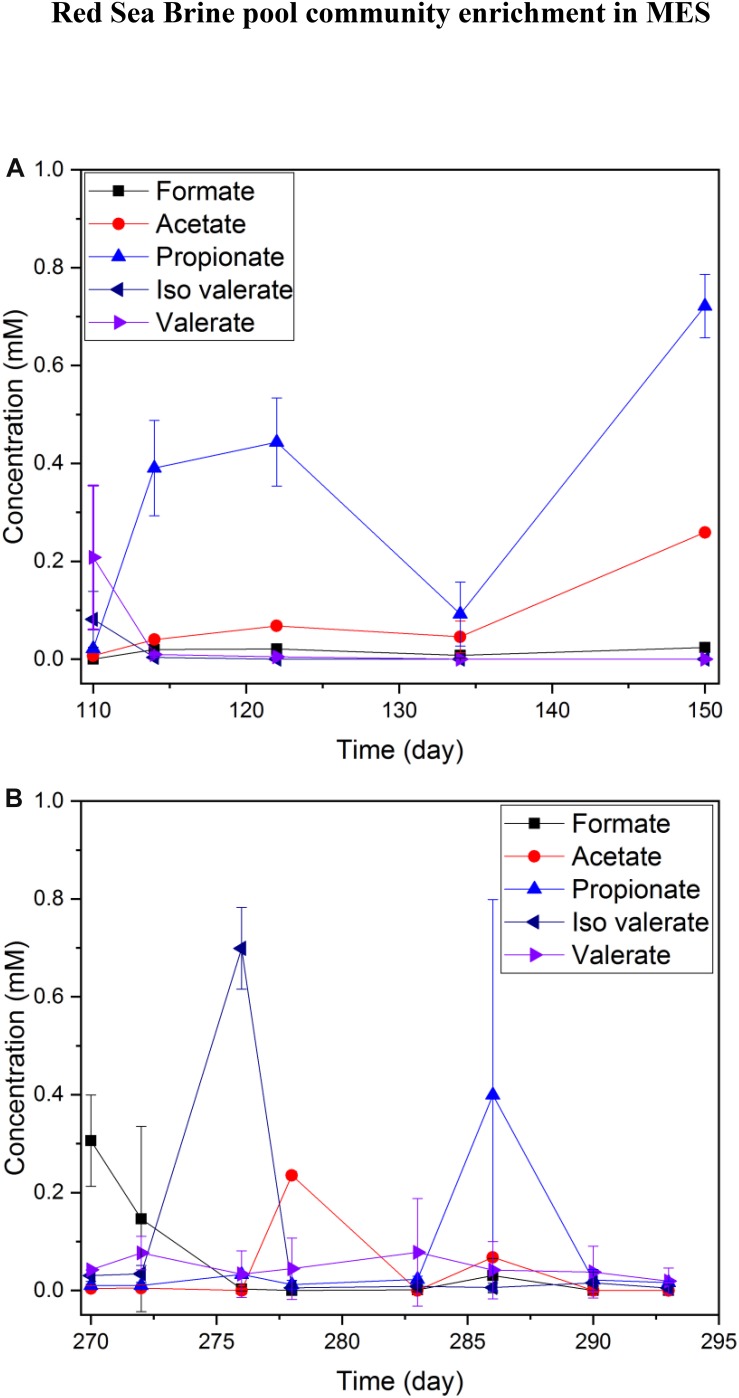
Average VFA production of duplicate MES reactors (R1 and R2) in phase I during a batch of operation with **(A)** brine pool solution (25% salinity) and **(B)** synthetic saline media (10% salinity). The data presented in **(A,B)** are based on one batch of operation, which is representative of several reproducible batches of operation.

Hydrogen production was the dominant process at the cathode. The VFAs produced from CO_2_ reduction were very low (<1 mM) in both 25% (Figure 4A) and 10% (Figure 4B) salinity. The highest observed VFA was propionate (reached 0.6 mM on day 11 and 0.85 mM on day 40 of a batch) in MES reactors operated under 25% salinity (Figure 4A), but trace amounts of acetate, valerate and iso-valerate were measured in both 25 and 10% salinity. The VFAs produced in MES reactors operated under 25% salinity were mainly limited to short chain carbon molecules (Figure 4A).

In a number of batches of MES operation with the replacement of new catholyte, no steady accumulation of acetate or any other VFA was observed in the catholyte. However, random peaks of VFAs such as acetate, iso-valerate were observed multiple times. Fast conversion to other unmeasured organics or product consumption by microbes for maintaining their metabolism might have occurred. Oxygen (generated from oxygen evolution reaction) intrusion from the anode to the cathode might be another possibility for the disappearance of some of the VFAs due to aerobic oxidation by heterotrophs, and this process promotes the growth of heterotrophs in the catholyte. Oxygen evolution at the anode occurred in the abiotic reactor operated with 10% synthetic saline media. The dissolved oxygen (DO) of anolyte from oxygen evolution reaction was measured at 1.93 mg/L, while the DO of the catholyte was 1.67 mg/L over 6 days of operation. Detection of DO in catholyte over the period of reactor operation implies that DO intrusion may have occurred from anolyte to catholyte through the Nafion membrane during the MES operation. In MES system, oxygen evolution is the main product of water splitting at the anode and oxygen diffusion from the anode to the cathode cannot be avoided using a proton exchange membrane such as Nafion. Oxygen diffusion across Nafion membrane has already been reported in the literature (Huang et al., 2018). Further, typical membranes used in BESs such as Nafion, anion exchange membrane or cation exchange membrane allow diffusion of oxygen (Kim et al., 2007).

### Enrichment of Biocathode From Phase I in Serum Vials Under H_2_:CO_2_ (80:20)

**FIGURE 5:**
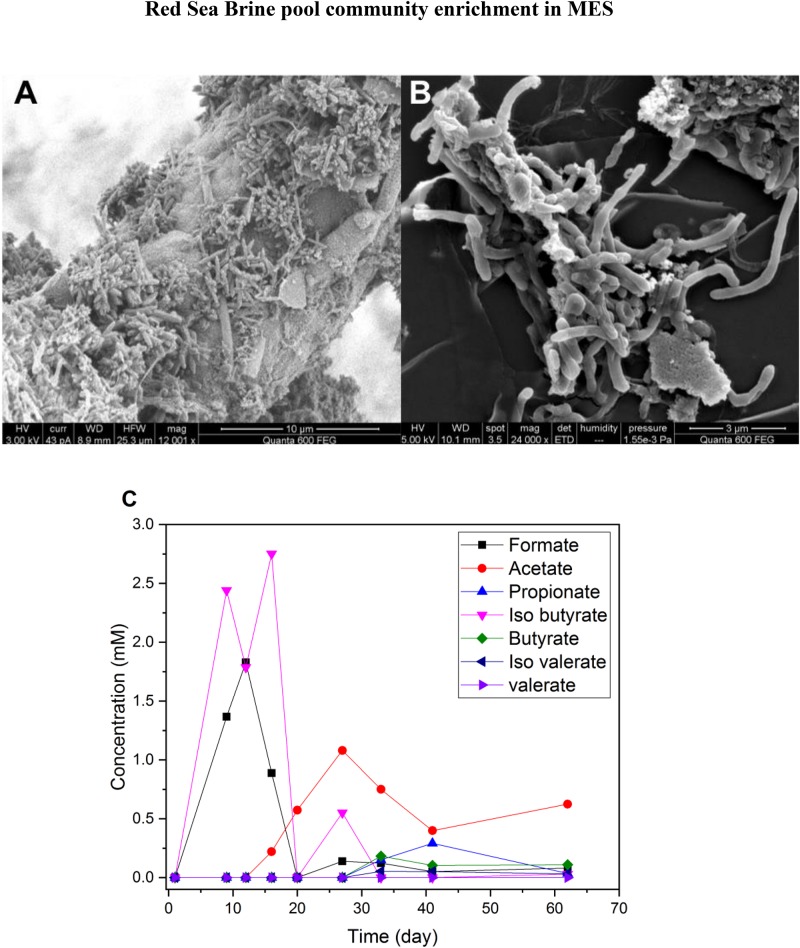
**(A)** SEM image of old biofilm (150 days) on the cathode surface from MES reactor R1 (phase I). **(B)** SEM image of biomass pellet obtained from H_2_:CO_2_ serum vial (phase I) after 60 days of incubation in synthetic saline media (10% salinity). **(C)** VFA production in H_2_:CO_2_ serum vial (phase I) with synthetic saline media (10% salinity).

On day 150, a piece of mature biocathode (Figure 5A) from R1 was inoculated into a serum vial containing synthetic saline media (10% salinity) and was incubated under H_2_:CO_2_ (80:20). Bacterial cells and clumps were seen on the SEM images of biomass pellet after culturing in H_2_:CO_2_ serum vials (Figure 5B). Higher production of VFAs, mainly formate, acetate and iso-butyrate, was observed in the serum vials (Figure 5C) compared to MES reactors R1 and R2 operated at 10% salinity (Figure 4B). In the first 12 days of incubation, formate production was observed which reached to slightly higher than 1.5 mM and after day 12, formate concentration declined and acetate started to appear which reached ∼1 mM on day 28. Acetate is the main product of CO_2_ reduction in Wood–Ljungdahl pathways with Formate as an intermediate. In Wood–Ljungdahl pathway, formate is first formed as intermediate product of CO_2_ reduction by formate dehydrogenase enzyme (Ljungdahl, 1986). It was apparent that the biocathode in the serum vial culture utilized H_2_ as electron donor and CO_2_ as electron acceptor as there was no other electron donor and acceptor (e.g., O_2_, sulfate, nitrate or metal ions). Also, during the first 20 days of incubation, up to 2.5 mM iso-butyrate was measured in the culture media, possibly due to amino acid fermentation occurring from anaerobic degradation of biomass (Harwood and Canale-Parola, 1982), but it was not detected after day 30.

### MES Operation in Phase II

**FIGURE 6:**
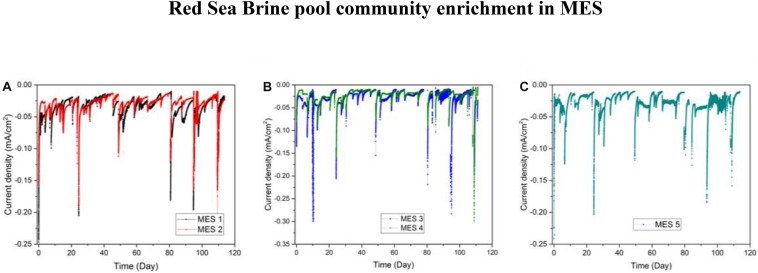
Chronoamperometry data during phase II of operation for **(A)** MES reactors (MES 1 and 2) inoculated with enriched biocathode from phase I MES reactors (i.e., R1 and R2) and operated using synthetic saline media (1% salinity), **(B)** MES reactors (MES 3 and 4) inoculated with enriched culture from H_2_:CO_2_ serum vial (phase I) and operated using synthetic saline media (10% salinity), and **(C)** MES reactor 5 inoculated with enriched culture from H_2_:CO_2_ serum vial (phase I) and operated using synthetic saline media (3.5% salinity).

In phase II of the experiment, secondary biofilms on new cathodes were established by inoculating phase I biocathodes (Inoculum_Biofilm, 10% salinity) into duplicates MES 1 and 2 (10% salinity) and by inoculating H_2_:CO_2_ enriched biomass (Inoculum_Culture, 10% salinity) into duplicates MES 3 and 4 (10% salinity) and separately into MES 5 (3.5% salinity). The current densities of MES reactors operated with 10% synthetic saline solution fluctuated between −0.01 and −0.05 mA/cm^2^ (Figures 6A,B). Current density in MES 1 and 2 were slightly higher in phase II (−0.03 ± 0.02 mA/cm^2^) than phase I (0.018 ± 0.007 mA/cm^2^). There was no considerable difference in current densities between MES 1 and 2 (Figure 6A) and MES 3 and 4 (Figure 6B). The MES 5 reactor operated with 3.5% salinity showed similar current consumption (−0.032 ± 0.02 mA/cm^2^) (Figure 6C) as MES 3 and 4 operated with 10% salinity. Based on the current density profiles, it can be inferred that the mode of inoculation (i.e., using previously enriched biocathode or enriched culture in serum vial) did not affect the current profile in MES.

**FIGURE 7:**
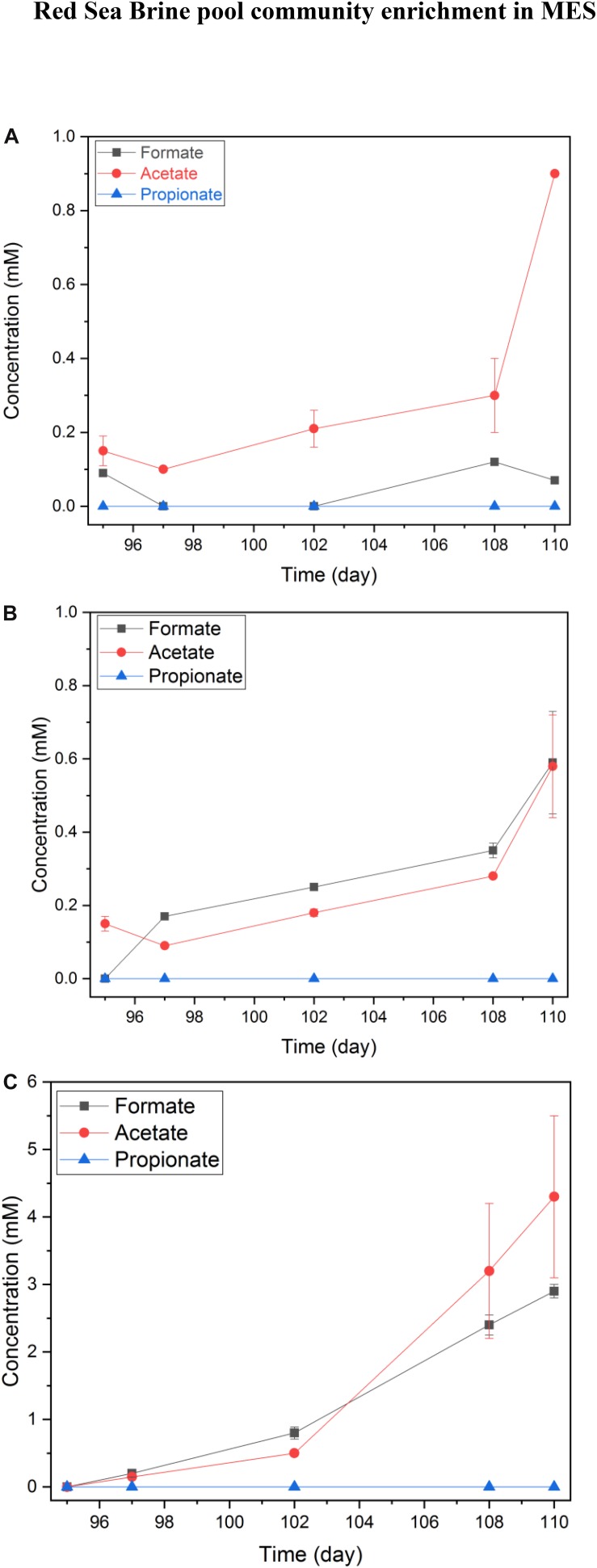
Average short-chained VFAs concentrations in MES reactors during phase II of operation. **(A)** average of duplicate MES reactors (MES 1 and 2) inoculated with enriched biocathode from phase I MES reactors (i.e., R1 and R2) and operated using synthetic saline media (10% salinity), **(B)** average of duplicate MES reactors (MES 3 and 4) inoculated with enriched culture from H_2_:CO_2_ serum vial (phase I) and operated with 10% using synthetic saline media (10% salinity), and **(C)** average of two batches for MES reactor (MES 5) inoculated with enriched culture from H_2_:CO_2_ serum vial (phase I) and operated using synthetic saline media (3.5% salinity). The data in **(A,B)** are based on one batch of operation, which is representative of several reproducible batches of operation.

The VFA concentrations profile in phase II was distinct from phase I, since a product accumulation trend was noticeable. Also, selectivity of the product generated, mainly acetate with formate as an intermediate, was observed in phase II (Figure 7). In a representative 15 day’s batch operation, the accumulation of formate and acetate was observed in MES 1 and 2 (Figure 7A) as well as MES 3 and 4 (Figure 7B). In MES 1 and 2, acetate concentration reached ∼1 mM in 15 days (Figure 7A) whereas in MES 3 and 4, acetate concentration was slightly higher than 0.6 mM after 15 days of batch operation but at the same time, formate concentration was slightly higher than 0.6 mM (Figure 7B). It should be noted that hydrogen evolution at the cathode was prominent at −1 V vs. Ag/AgCl cathode potential.

Remarkably, the concentrations of VFAs increased in MES 5, which was operated at 3.5% salinity with acetate concentration during a batch reaching ∼4 mM (Figure 7B). Hydrogen gas, formate, and acetate were accounted as the main products in MES 5 at −1 V vs. Ag/AgCl imposed cathode. The hydrogen measured in the headspace of MES reactors in mmole electron equivalent was 12.01 for MES 1 and 2, 9.85 for MES 3 and 4, and 10.50 for MES 5 during the 15 days of operation. These results suggest that VFA production from CO_2_ reduction appeared to be more compatible at 3.5% salinity. When the salinity decreased from 25 to 10% and further down to 3.5%, VFA production was enhanced, possibly due to increased solubility of CO_2_ with decrease in the salinity of the media. At the same time, the salinity stress for the biocathode community was also reduced. These factors could have promoted the accumulation of products in the catholyte from CO_2_ reduction. Based on the products formation (Figure 7), hydrogen production, and current densities (Figure 6), the coulombic efficiency (CE) for MES 1 and 2 (10% salinity), MES 3 and 4 (10% salinity), and MES 5 (3.5% salinity) was 63, 72, and 86%, respectively. Overall, VFA production and accumulation was more pronounced in phase II than phase I of MES operation due to the enrichment of a CO_2_ reducing microbial community.

### Biocathode Microbial Community Composition in Phase I and II

**FIGURE 8:**
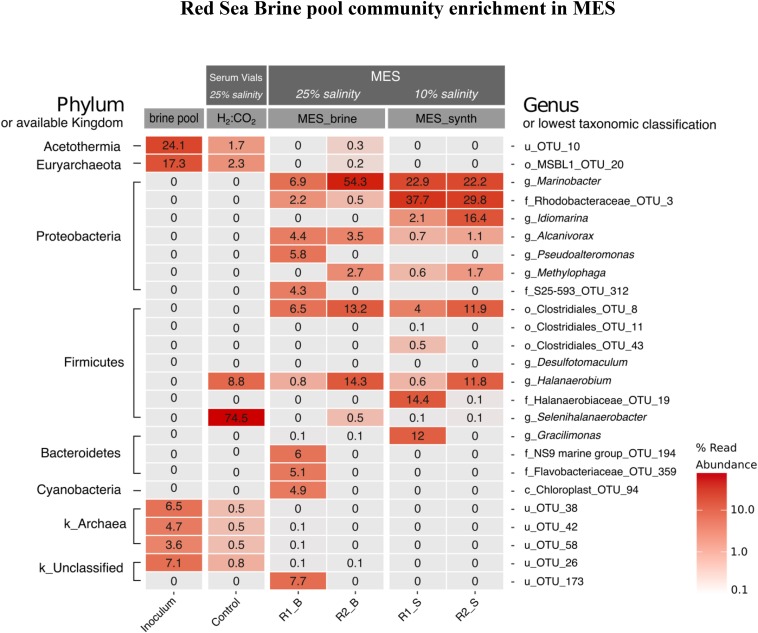
Heatmap distribution of the 25 most abundant phylotypes across all samples in phase I (see Figure 1). The taxa level shown on the left-hand side represents the phyla while on the right-hand side represents the lowest classification level possible (u: unclassified, c: class, o: order, f: family, or g: genus). The scale in the bottom right represents relative abundance (%) of reads. B refers to brine pool and S refers to synthetic saline media.

A heatmap of the top 25 OTUs of each sample in phase I with relative abundance ≥ 0.1% is presented in Figure 8. There was a shift in brine pool microbial community with the adaptation toward CO_2_ reducing cathodic environment of MES reactors (R1 and R2) and also after H_2_:CO_2_ [80:20] (control 1) cultivation. The brine pool sediment inoculum was predominated by the candidate phylum Acetothermia (24%) and the order MSBL1 (Mediterranean Sea Brine Lakes 1) (17.3%), which belongs to the phylum Euryarchaeota. Acetothermia is one of the groups that have been detected in hypersaline environments (Nigro et al., 2016) and its capability of acetogenesis via Wood–Ljungdahl (reductive Acetyl-CoA) pathway of CO_2_ fixation was predicted by genome-resolved metagenomic analysis (Takami et al., 2012). The presence of Acetothermia in the Red Sea brine pool suggests acetogenic lifestyle in these environments. MSBL1 was previously described from other Red Sea brine pools (Mwirichia et al., 2016). A long-term enrichment of brine pool community in MES resulted in the disappearance of Acetothermia and MSBL1 with Proteobacteria and Firmicutes becoming dominant phyla in the MES reactors (Figure 8). This shift in the brine pool community composition occurred due to the selective pressure of the cathodic environment in the MES reactors. Similarly, Acetothermia and MSBL1 from the original brine pool significantly decreased in relative abundance when incubated in serum vial under H_2_:CO_2_ (control 1). Firmicutes were significantly enriched under H_2_:CO_2_ (control 1) and 25% salinity with *Selenihalanaerobacter* (phylum Firmicutes, order Halanaerobiales) accounting for 74.5% of the total reads.

Proteobacteria was the dominant phylum at the biocathode of R1 and R2 operated at 25% salinity (brine pool) and later at 10% salinity (synthetic saline media). In MES reactor R1 operated with brine pool as electrolyte (i.e., R1_B in Figure 8), the dominant genus was *Marinobacter* (6.9% of sequence reads) which reached 22.9% in synthetic saline media (R1_S in Figure 8). Likewise, the genus *Marinobacter* was dominant member at the biocathode of MES reactor R2 with relative abundance of 54.3% using brine pool as electrolyte (R2_B in Figure 8) and 22.3% using saline synthetic media (R2_S in Figure 8). Firmicutes was the second dominant phylum in the MES reactors with obligate anaerobes belonging to the order Clostridiales_OTU_8 (6.5% in R1_B and 13.2% in R2_B) and genus *Halanaerobium* (14.3% in R2_B and 11.8% in R2_S).

**FIGURE 9:**
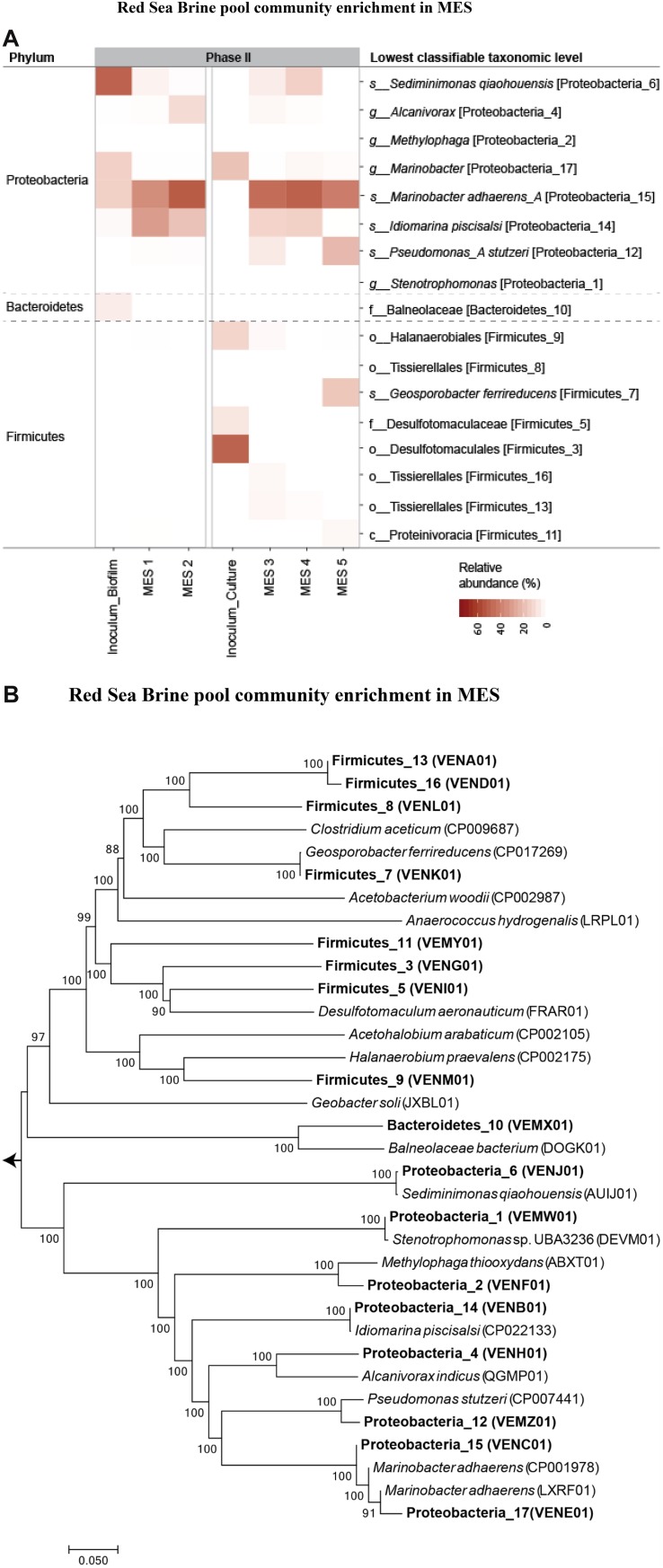
**(A)** Relative abundance heatmap plot of MAGs. The taxa level shown on the left-hand side represents the phyla while on the right-hand side represents the lowest classification level possible (u: unclassified, c: class, o: order, f: family, or g: genus). **(B)** Neighbor-joining phylogenetic tree showing the evolutionary relationship between the 17 recovered MAGs and closely related genomes downloaded from the NCBI genome repository. The tree includes MAGs (shown in bold text) recovered from the different samples. GenBank accession numbers of the genomes are provided in parenthesis. Branch node numbers represent bootstrap support values and the bootstrap consensus inferred from 1000 iterations.

For further microbial community characterization, MAG approach was used to extract 17 high-quality draft genomes or MAGs (Supplementary Table S1) from the different samples in phase II, including the inoculum sources from phase I that were used for seeding the MES reactors in phase II. The recovered 17 population genomes accounted for 87 ± 4% of the quality filtered sequence reads, indicating that the sequencing depth was sufficient to obtain a comprehensive insight of the microbial community structure in these systems. Hereafter, we will refer to these 17 organisms by their phyla and a number as MAG IDs mentioned in Supplementary Table S1. The genome sequences in the MES reactors belonged to two main phyla: Proteobacteria and Firmicutes (Figure 9). A heatmap representation of the relative abundance of the 17 MAGs is presented in Figure 9A. The taxonomic affiliation of the genome bins revealed that Proteobacteria_15 was the dominant bin (46 ± 7% of total reads) in all the MES reactors (Figure 9A) and based on the phylogenomic tree (Figure 9B) this bin was found to be closely related (96.6% nucleotide-level genomic similarity) to *Marinobacter adhaerens*. An average nucleotide identity (ANI) cutoff score > 95% suggests that a given pair of genomes belongs to the same species (Figueras et al., 2014). In the current study, ANI was calculated using ANI calculator^2^. Proteobacteria_14 (97.7% nucleotide-level genomic similarity to *Idiomarina piscisalsi*) was the second dominant bin (20 ± 8% of total reads) in MES reactors operated at 10% salinity. In contrast, Proteobacteria_12 (97.8% nucleotide-level genomic similarity to *Pseudomonas stutzeri*) was the second dominant bin (22% of total reads) after *Marinobacter* in MES 5 operated at 3.5% salinity. In phase II of MES operation, the biocathode community in all the MES reactors showed increase in the relative abundance of Proteobacteria_15 compared to the inocula from phase I (i.e., Inoculum_Biofilm and Inoculum_Culture) that were used to seed these reactors (Figure 9A). In contrast, Proteobacteria_17 (95.8% nucleotide-level genomic similarity to *M. adhaerens*) decreased in relative abundance compared to the inocula. The inoculum source (i.e., Inoculum_Biofilm) for MES 1 and 2 had high relative abundance (50%) of Proteobacteria_6 (97.4% nucleotide-level genomic similarity to *Sediminimonas qiaohouensis*), Proteobacteria_15 (15%) and Proteobacteria_17 (15%), whereas the inoculum source (Inoculum_Culture) for MES 3 to 5 had high relative abundance of Proteobacteria_17 (19%), Firmicutes_3 (50%), Firmicutes_5 (8%) and Firmicutes_9 (13%).

Both amplicon sequencing and metagenomic analysis showed that members of the genus *Marinobacter* were dominant at the biocathode of MES. The high relative abundance of Proteobacteria_15 (most closely related to *M. adhaerens*) in MES 3 to 5 (47 ± 5% of total reads) compared to serum vial Inoculum_Culture (0.64% of total reads) suggests some role of cathodic environment in their enrichment. No significant change in biocathode Proteobacteria composition was observed when comparing biofilm inoculated MES reactors (MES 1 and 2) with serum vial biomass inoculated MES reactors (MES 3 and 4), suggesting that both inoculum sources converged to similar Proteobacteria community due to the strong selective pressure of the cathodic environment in MES.

As for Firmicutes, their relative abundance was significantly higher in Inoculum_Culture and MES 3 to 5 compared to Inoculum_Biofilm and MES 1 and 2. The biocathode community in MES 5 (3.5% salinity), which showed the highest acetate production (Figure 7C), had the highest relative abundance of Firmicutes (23% of total reads) compared to MES 1 to 4 operated at 10% salinity, with Firmicutes_7 (99.6% nucleotide-level genomic similarity to *Geosporobacter ferrireducens*) being the dominant (18%) Firmicutes (Figure 9). Firmicutes_7 was not detected in Inoculum_Culture, Inoculum_Biofilm and MES reactors operated at 10% salinity (i.e., MES 1 to 4). It should be noted that the majority of genomes belonging to Firmicutes in the current study represent novel Firmicutes with no cultured or sequenced representatives (Figure 9), emphasizing the lack of knowledge on the microbial community of Firmicutes in these hypersaline environments.

### Presence of Genes Encoding Enzymes for CO_2_ Fixation Pathways, Hydrogenases, and Formate Dehydrogenases

**FIGURE 10:**
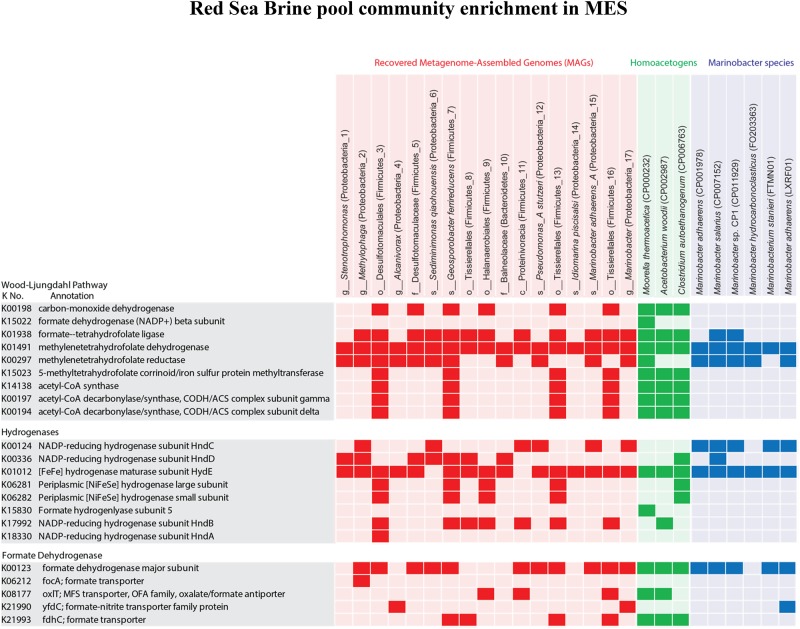
Metabolic comparison of the 17 recovered MAGs with well-known homoacetogens and several closely related *Marinobacter* genomes. Marker genes involved in the biochemical CO_2_ fixation pathway (Wood–Ljungdahl pathway), hydrogenases and formate dehydrogenase in the different genomes were presented as presence (colored) and absence (blank). These genes were presented here with annotation and KEGG Orthology (KO) identifiers (or K numbers). GenBank accession numbers of the reference genomes are provided in parenthesis.

Since members of the genus *Marinobacter* are heterotrophs (unable to fix CO_2_) (Onderko et al., 2019) and amplicon sequencing and metagenomics revealed that they are the dominant members at the biocathode (Figures 8, 9) where no other carbon source was provided other than CO_2_, we screened the MAGs of other members in the biocathode for the presence of genes encoding enzymes for CO_2_ fixation pathways: Wood–Ljundahl pathway, reductive citric acid cycle, 3-hydroxypropionate bicycle, hydroxypropionate-hydroxybutyrate cycle, and dicarboxylate-hydroxybutyrate cycle. Of the different CO_2_ fixation pathways, we only detected marker genes encoding enzymes of a complete Wood–Ljundahl pathway, including genes encoding for formyl-tetrahydrofolate ligase, which catalyzes the activation of formate utilizing ATP, methylene-tetrahydrofolate dehydrogenase/methenyl-tetrahydrofolate cyclohydrolase, 5,10- methylene-tetrahydrofolate reductase, 5-methy-tetrahydrofolate corrinoid/iron sulfur protein methyltransferase, acetyl-CoA synthase, carbon-monoxide dehydrogenase, and CO dehydrogenase/acetyl-CoA synthase (CODH/ACS) (Sewell et al., 2017). Genes for complete Wood–Ljundahl pathway were mainly detected in 4 (Firmicutes_3, Firmicutes_7, Firmicutes_13, and Firmicutes_16) out of the 17 MAGs (Figure 10). The presence of Firmicutes_7, Firmicutes_13, and Firmicutes_16 at the biocathode suggests that *Marinobacter* sp. most probably received fixed carbon through the activity of acetogenic Firmicutes. Based on genomic data we predict that these Firmicutes were mainly using the poised cathode directly or indirectly via H_2_ generated from the HER as the electron donor for driving the reductive Wood–Ljundahl pathway because of the presence of genes encoding for hydrogenases in their genomes, such as [NiFeSe]-hydrogenase (Croese et al., 2011; Deutzmann et al., 2015). Further, the presence of genes encoding for formate dehydrogenases and formate transporter in these Firmicutes suggest their capability of using formate as electron donor (Sewell et al., 2017) and possibly mediating formate-dependent uptake of electrons from the cathode (Reda et al., 2008; Croese et al., 2011).

## Discussion

The use of brine pool solution (25% salinity) as electrolyte in MES reactors during the initial enrichment in phase I was intended to mimic the natural brine pool environment as much as possible with the same micronutrients, salinity and metal ions. Since microbial density is, in general, low in brine pools (Shehab et al., 2017), a long-term enrichment in MES mode was adopted to allow acclimation of brine pool microbial community to autotrophic and cathodic condition. In succeeding stages of MES operation, the brine pool solution was replaced with synthetic saline media (10% salinity) to eliminate the probable influence of metals from the original brine to act as electron acceptors. The production of acetate (Figure 7) in the MES reactors at 10% salinity, with no other carbon source other than CO_2_ and H_2_ or cathode as the sole electron donor, suggests homoacetogenic activity of biocathode community. Lowering the salinity from 10 to 3.5% increased the concentration of acetate and formate in MES 5 (Figure 7C). This increase in homoacetogeneis at 3.5% salinity could be due to the increase in CO_2_ dissolution and at the same time, reduction of salinity stress on homoacetogens. Life at haloalkaline conditions is energetically costly, and therefore, extreme haloalkaliphiles could hardly produce metabolites (Oren, 2011). Energy conservation at high salinity may limit the VFA production from CO_2_ reduction in homoacetogens. At high salinity, microbes produce organic compatible solutes, also known as osmoprotectants, such as ectoine, glycine, betaine or have high intracellular K^+^ concentrations (Shivanand and Mugeraya, 2011), that can help bacteria to provide the necessary osmotic balance under high saline condition. Acetate and ${\text{HCO}}_{3}^{-}$ assimilation to produce ectoine for osmotic adaptation was also reported in haloextremophiles (Peters et al., 1990; Pastor et al., 2010). In homoacetogens, acetyl-CoA formed from CO_2_ reduction is converted to acetate during catabolism and the same acetyl-CoA is converted to cell carbon during anabolism (Diekert and Wohlfarth, 1994). Thus, it is expected that cell biomass production be more dominant than acetate production in the MES reactors operated at high salinity (25 and 10%). Interestingly, Firmicutes_7 (closely related to *G. ferrireducens*) was only detected in MES 5 (3.5% salinity), seeded with the same inoculum (Incoculum_Culture) as MES 3 and MES 4, which were operated at 10% salinity. *G. ferrireducens* is a known halophilic homoacetogen (Jung et al., 2018) and it has been reported that *G. ferrireducens* does not require NaCl for its growth and can grow up to 4% (w/v) NaCl (Hong et al., 2015). This could explain why Firmicutes_7 was significantly enriched after the salinity was lowered to 3.5% (seawater salinity) in MES 5 biocathode, supporting the high acetate production. In contrast, *Marinobacter* sp. (closely related to *M. adhaerens*) remained equally abundant at 10 and 3.5% salinity. *M. adhaerens* can tolerate a wide range of saline conditions (0.5–20%) (Kaeppel et al., 2012). It should be noted that methanogens were not detected in the MES reactors and serum vials in both phases of operation and there was no detection of CH_4_ suggesting that the environmental conditions in the cathode were not suitable for their enrichment.

The concentrations of acetate was higher in the serum vials operated under H_2_:CO_2_ (80:20) and 10% salinity (Figure 5C) compared to MES reactors operated at 10% salinity (Figures 7A,B). Using carbon cloth as cathode material, the current densities recorded in the MES reactors were low (Figures 2, 6) even at −1 V vs. Ag/AgCl. This obviously relates to the low production of H_2_ via HER resulting in the low production of VFAs from CO_2_ reduction by acetogenic Firmicutes. In contrast, H_2_ was abundant in the serum vials. Improved cathode material for HER is required to increase the product yield under saline conditions. Furthermore, CO_2_ dissolution is expected to be low under highly saline solutions (Carvalho et al., 2015). Lower dissolved CO_2_ affects the microbial CO_2_ reduction and also can limit the proton availability (carbonic acid formation) thereby affecting the HER rate.

Incubation in serum vials under H_2_:CO_2_ (80:20) environment enhances the growth of CO_2_ reducers and can lead to an increase in their biomass density. However, the microbial communities become H_2_-dependent and may not acquire any electrical interaction with the cathode. This limitation can be eliminated by directly enriching the community at the cathode of MES. The electrically poised cathode in MES can provide multiple route of electron transfer (through direct uptake from cathode and/or through H_2_ produced by HER at the cathode surface). Moreover, MES cathode environment may create a niche environment to select for cathode-driven homoacetogenic lifestyle and electrochemically active bacteria. Using the same inoculum source (brine pool sediment and solution), different microbial communities were enriched under MES environment compared to incubation in serum vials (control 1) under H_2_:CO_2_ (80:20) and 25% salinity. *Marinobacter* sp. (closely related to *M. adhaerens*) was the dominant biocathode community detected in the MES reactors (Figure 8), whereas the genera *Selenihalanaerobacter* and *Halanaerobium* from the phylum Firmicutes (order Halanaerobiales) were dominant in H_2_:CO_2_ serum vials. The order Halanaerobiales contains halophilic anaerobes with fermentative or homoacetogenic metabolism (Blum et al., 2001). The enrichments of homoacetogens belonging to the phylum Firmicutes in H_2_:CO_2_ serum vial culture were mostly reported from non-saline sources (Ragsdale and Pierce, 2008). *Marinobacter* sp. remained dominant at the biocathode of MES reactors after replacing the brine pool solution (25% salinity) with synthetic saline media (10% salinity) (Figure 8). Predominance of *Marinobacter* sp. in the biocathode was continued in phase II of MES operation at 10 and 3.5% salinity (Figure 9). The high enrichment of *Marinobacter* sp. in MES reactors and their lack of enrichment in the serum vials (control 1) under H_2_:CO_2_ (80:20) suggest some role of cathodic environment in their enrichment, which is discussed further below. It should be noted that the microbial community composition of Firmicutes enriched in the serum vial (Inoculum_Culture) under H_2_:CO_2_ (80:20) in phase I was different from the composition of Firmicutes enriched at the biocathode of MES in phase II (Figure 9), also suggesting some role of cathodic environment in their enrichment.

**FIGURE 11:**
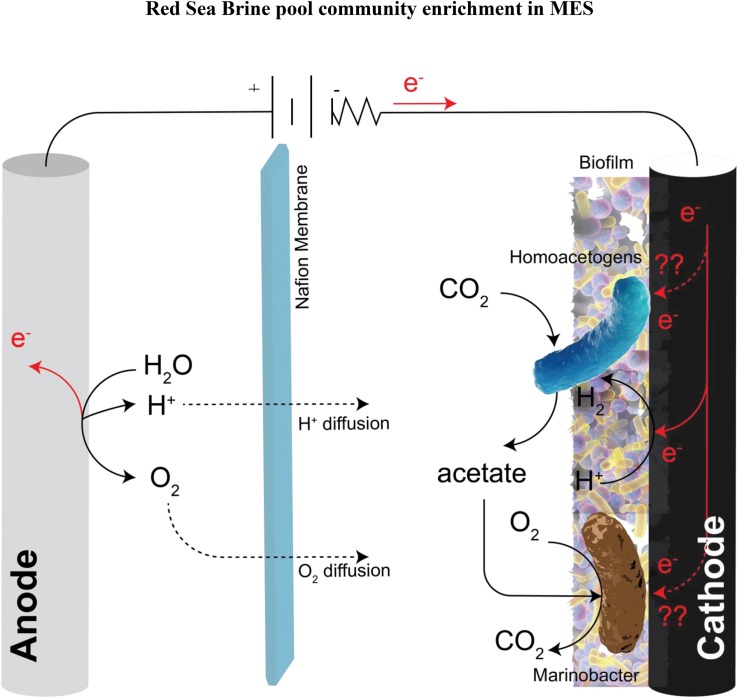
Schematic of the potential metabolic interactions of the cathodic community with CO_2_ as the only carbon source provided and the cathode as the primary electron donor. These metabolic interactions were inferred based on genome-resolved metagenomic analysis and electrochemical data. In the schematic electrons are taken from the cathode to generate H_2_ abiotically through the HER. Homoacetogens from the phylum Firmicutes utilize the abiotic H_2_ via hydrogenases as a source of electron donor for driving the reductive Wood–Ljundahl pathway for CO_2_ fixation to acetate. *Marinobacter* sp. utilize the fixed carbon (acetate) as their electron donor and carbon source using oxygen that diffuses from the anode as the electron acceptor.

*Marinobacter* sp. are considered “biogeochemical opportunitrophs” (Singer et al., 2011) because they have the ability to utilize a wide variety of substrates and are versatile in respiration modes; they could be aerobic or facultative anaerobe depending on the strain (Handley and Lloyd, 2013). They have the ability to shift their metabolic activity in the presence of O_2_ gradient and also, they can reduce nitrate presumably to nitrite and several species have been reported to utilize nitrate as an electron acceptor (Handley and Lloyd, 2013; Bird et al., 2018). Fermentation of glucose in the presence of nitrate was observed in *Marinobacter* sp. (Nakano et al., 2010). Likewise, iron oxidation capability of *Marinobacter* sp. has been reported (Singer et al., 2011). Their abundance at the anode (Monzon et al., 2015, 2017; Rousseau et al., 2016) and cathode (Strycharz-Glaven et al., 2013; Debuy et al., 2015; Rowe et al., 2015; Wang et al., 2015; Stepanov et al., 2016) biofilm consortium in BESs was also been reported. Recently, *Marinobacter atlanticus* CP1 was reported to be the dominant member of the biocathode community in MFC with CO_2_ as the only carbon source and no electron donor other than the poised cathode (Strycharz-Glaven et al., 2013; Wang et al., 2015). The authors hypothesized that “*Candidatus* Tenderia electrophaga,” an electroautotroph, catalyze the electron transfer from the cathode to oxygen and CO_2_ for growth. “*Ca.* Tenderia electrophaga” fixes CO_2_ through the Calvin-Bensen-Bassham cycle and *M. atlanticus* CP1 presumably receives fixed carbon from “*Ca.* Tenderia electrophaga.” In the current study, we proposed based on genome-resolved metagenomic analysis and electrochemical data a hypothetical model of metabolic interactions at the biocathode biofilm in MES with no added carbon other than CO_2_ and poised cathode as the primary electron donor (Figure 11). We hypothesized that the biocathode biofilm was fixing CO_2_ through the activity of Firmicutes to support the growth of *Marinobacter* sp. The cathode was used as the electron donor, directly or indirectly via H_2_, to support the growth of homoacetogens (phylum Firmicutes) by reducing CO_2_ to acetate as the catabolic end product, which was then utilized by *Marinobacter* sp. and other aerobic heterotrophs for growth through aerobic respiration. The finding that *Marinobacter* can live in close association with homoacetogens in *ex situ* condition is a unique finding and was attained only through enrichment in MES biocathode. This finding suggests that in environments that are limited with organics, *Marinobacter* spp. can thrive with homoacetogens which can provide them with fixed carbon.

The presence of *Marinobacter* sp. in the anode and cathode biofilm of BES suggest some electrochemical interaction between *Marinobacter* spp. and electrodes. The genomes of *Marinobacter* spp. (Proteobacteria_15 and Proteobacteria_17) revealed the presence of genes for multiheme c-type cytochromes that may participate in extracellular electron transfer (EET), however, they do not contain large multiheme c-type cytochromes similar to the ones present in known electrochemically active bacteria like *Geobacter sulfurreduces* and *Shewanella oneidensis* MR1 (Onderko et al., 2019). Recently, it was demonstrated that *M. atlanticus* CP1 biofilms can generate very low level of anodic and cathodic current in BES fed with oxygenated seawater medium supplemented with succinate as carbon and electron donor source, suggesting its ability to perform EET (Onderko et al., 2019). Addition of redox-active species such as riboflavin or excess trace minerals resulted in an increase in current production indicating that soluble redox mediators can facilitate EET in *M. atlanticus* CP1. Future studies are needed to better understand the EET mechanism in *Marinobacter* sp.

## Conclusion

Our study provided evidence for the enrichment of *Marinobacter* sp. and halophilic homoacetogens at the biocathode of MES system using Red Sea brine pool as inoculum. Here we demonstrated that with no carbon source other than CO_2_, and with poised cathode as the main electron donor, *Marinobacter* sp. was the dominant biocathode community in MES. Their dominance at the cathode was possible due the presence of O_2_ (electron acceptor) escaping from the anode to the cathode and fixed CO_2_ (carbon and energy source) from the activity of homoacetogenic Firmicutes, which utilized the poised cathode as electron donor to reduce CO_2_ to acetate. These findings obtained using MES system might have important implications for how *Marinobacter* spp. may interact with CO_2_ fixing bacteria such as homoacetogens in natural saline environments under organic-carbon limiting conditions.

## Data Availability Statement

All sequencing data associated with this project are available at NCBI under BioProject PRJNA545216. The MAG sequences were submitted to DDBJ/ENA/GenBank. The MAG IDs and their accession numbers are given in Supplementary Table S1.

## Author Contributions

KK and PS conceptualized and designed the experiments. GM and DD sampled the brine pool in the Red Sea. MFA performed the experiments. MFA, KK, PS, MA, and SB analyzed the data. SB, MFA, and PS wrote the manuscript. AR helped in amplicon sequencing analysis and preparation of figures. MA helped in metagenomic analysis and preparation of figures. KK, PS, and MA helped in thoughtful discussion and comprehensively revising the manuscript. All co-authors revised the manuscript.

## Conflict of Interest

The authors declare that the research was conducted in the absence of any commercial or financial relationships that could be construed as a potential conflict of interest.

## Footnotes

1 http://merenlab.org/2016/06/22/anvio-tutorial-v2/

2 http://enve-omics.ce.gatech.edu/ani/index
