# Supplementary material for: Enrichment of Marinobacter sp. and Halophilic Homoacetogens at the Biocathode of Microbial Electrosynthesis System Inoculated With Red Sea Brine Pool
Source: Front Microbiol. 2019 Nov 7;10:2563. doi: 10.3389/fmicb.2019.02563 (PMC6855130; doi:10.3389/fmicb.2019.02563)
Supplement: Supplementary file 1 [file Data_Sheet_1.PDF]

Supplementary Data

Enrichment of *Marinobacter* sp. and Halophilic Homoacetogens at the Biocathode of Microbial Electrosynthesis System Inoculated with Red Sea Brine Pool

Manal F. Alqahtani[1§], Suman Bajracharya[1§], Krishna P. Katuri[1], Muhammad Ali[1], Ala'a Ragab[1], Grégoire Michoud[2], Daniele Daffonchio[2] and Pascal Elias Saikaly[1,*]

[1]King Abdullah University of Science and Technology, Water Desalination and Reuse Center, Biological and Environmental Science and Engineering Division, Thuwal 23955-6900, Saudi Arabia

[2]King Abdullah University of Science and Technology, Red Sea Research Center, Biological and Environmental Science and Engineering Division, Thuwal 23955-6900, Saudi Arabia

[§]These authors contributed equally to this paper and should be considered as co-first authors

*** Correspondence:**
Prof. Pascal Saikaly (pascal.saikaly@kaust.edu.sa)

Materials and Method

16S rRNA gene library preparation

The extracted DNA (up to 10 ng) was used as a template for PCR amplification. Each PCR reaction (25 µL) consisted of dNTPs (100 µM of each), $MgSO_4$ (1.5 mM), Platinum Taq DNA polymerase HF (0.5 U/reaction), Platinum High Fidelity buffer (1X) (Thermo Fisher Scientific, USA) and tailed primer mix (400 nM of each forward and reverse primer). PCR was run with the following program: Initial denaturation at 95° C for 2 min, 35 cycles of amplification (95°C for 20 s, 50°C for 30 s, 72°C for 60 s) and a final elongation at 72°C for 5 min. Duplicate PCR reactions were performed for each sample and the duplicates were combined after PCR. The forward and reverse tailed primers were designed according to Illumina and have primers targeting the Archaea and Bacteria 16S rRNA gene region V4: forward primer [515FB] GTGYCAGCMGCCGCGGTAA and reverse primer [806RB] GGACTACNVGGGTWTCTAAT (Apprill et al. 2015). The primer tails enable attachment of Illumina Nextera adaptors, which is required for sequencing in a subsequent PCR. The resulting amplicon libraries were cleaned by following the standard protocol for Agencourt Ampure XP Beads (Beckman Coulter, USA) with a bead to sample ratio of 4:5. DNA was eluted in 25 µL of nuclease free water (Qiagen, Germany). DNA concertation was estimated using Qubit dsDNA HS/BR Assay kit (Thermo Fisher Scientific, USA). Gel electrophoresis using Tapestation 2200 and Genomic DNA screentapes (Agilent, USA) were performed to examine product size and purity of a subset of sequencing libraries.

Sequencing libraries were prepared from the purified amplicon libraries using a second PCR. Each PCR reaction (25 µL) contained PCRBIO HiFi buffer (1x), PCRBIO HiFi Polymerase (1 U/reaction) (PCRBiosystems, UK), adaptor mix (400 nM of each forward and reverse) and up to 10 ng of amplicon library template. PCR was conducted with the following program: Initial denaturation at 95°C for 2 min, 8 cycles of amplification (95°C for 20 s, 55°C for 30 s, 72°C for 60 s) and a final elongation at 72° C for 5 min. The resulting sequencing libraries were purified using the standard protocol for Agencourt Ampure XP Beads (Beckman Coulter, USA) with a bead to sample ratio of 4:5. DNA was eluted in 25 µL of nuclease free water (Qiagen, Germany). DNA concentration was measured using Qubit dsDNA HS Assay kit (Thermo Fisher Scientific, USA). Gel electrophoresis using Tapestation 2200 and D1000/High sensitivity D1000 screentapes (Agilent, USA) was used to validate product size and purity of a subset of sequencing libraries.

Table S1. Characteristics of Metagenome-Assembled Genomes (MAGs).

MAG ID	Taxon[a]	MAG size (bp)	No. contigs	N50[b]	Completeness (%)	Contamination (%)	Accession
Proteobacteria_1	g__*Stenotrophomonas*	4071812	46	141137	99.6	0.8	VEMW01
Proteobacteria_2	g__*Methylophaga*	2869380	31	221470	99.6	0.4	VENF01
Firmicutes_3	o__Desulfotomaculales	4298674	48	160835	99.3	1.0	VENG01
Proteobacteria_4	g__*Alcanivorax*	3529875	21	875658	99.6	2.0	VENH01
Firmicutes_5	f__Desulfotomaculaceae	3168988	19	303033	100.0	1.9	VENI01
Proteobacteria_6	s__*Sediminimonas qiaohouensis*	3223358	37	159904	99.5	0.3	VENJ01
Firmicutes_7	s__*Geosporobacter ferrireducens*	5625643	34	307024	99.3	3.0	VENK01
Firmicutes_8	o__Tissierellales	4084979	36	293581	99.3	3.8	VENL01
Firmicutes_9	o__Halanaerobiales	4196602	52	198868	99.1	0.9	VENM01
Bacteroidota_10	f__Balneolaceae	4076035	99	62318	99.7	0.0	VEMX01
Firmicutes_11	c__Proteinivoracia	3089666	77	77223	95.3	0.0	VEMY01
Proteobacteria_12	s__*Pseudomonas_A stutzeri*	4415240	91	97157	98.7	0.8	VEMZ01
Firmicutes_13	o__Tissierellales	3560429	87	81442	95.6	1.4	VENA01
Proteobacteria_14	s__*Idiomarina piscisalsi*	2366869	38	153943	92.7	0.9	VENB01
Proteobacteria_15	s__*Marinobacter adhaerens*_A	4286748	31	323374	97.0	0.0	VENC01
Firmicutes_16	o__Tissierellales	3830044	180	33523	93.5	3.5	VEND01
Proteobacteria_17	g__*Marinobacter*	3988649	62	153758	67.6	0.0	VENE01

[a] The lowest classifiable taxonomy level (c: class, o: order, f: family, g: genus or s: species) determined by using the Genome Taxonomy Database (gtdb).
[b] The N50 value represents half of the genome contigs that are larger than or equal to this contig size.

Table S2. Chemical characterization of the brine pool interface solution.

**Component**	**Concentration**
Li (ppb)	120.8
Be (ppb)	1.605
Na (ppb)	1.54E+08
Mg (ppb)	2.82E+06
Al (ppb)	9.174
K (ppb)	2.66E+06
Ca (ppb)	6.60E+05
Sc (ppb)	3.823
Ti (ppb)	3.716
V (ppb)	21.26
Cr (ppb)	42.39
Mn (ppb)	849.9
Fe (ppb)	1.73E+03
Co (ppb)	<0.000
Ni (ppb)	<0.000
Cu (ppb)	130.9
Zn (ppb)	<0.000
As (ppb)	5.643
Rb (ppb)	110.4
Sr (ppb)	5.23E+03
Ag (ppb)	5.535
Cd (ppb)	<0.000
Ba (ppb)	14.23
Pb (ppb)	8.50E-01
NH_4-N(mg/L)	5.6
NO_2-N (mg/L)	0.037
NO_3-N (mg/L)	0.5
SO_4 (mM)	79.22
TDS (g/L)	234
TOC (mg/L)	1.029
pH	5.85

References

Apprill A, McNally S, Parsons R, Weber L (2015) Minor revision to V4 region SSU rRNA 806R gene primer greatly increases detection of SAR11 bacterioplankton . Aquat Microb Ecol 75:129–137
